# The decline of human endogenous retroviruses: extinction and survival

**DOI:** 10.1186/s12977-015-0136-x

**Published:** 2015-02-02

**Authors:** Gkikas Magiorkinis, Daniel Blanco-Melo, Robert Belshaw

**Affiliations:** Department of Zoology, University of Oxford, Oxford, OX1 3PS UK; Virus Reference Department, Microbiology Services, Public Health England, Colindale, London, UK; Department of Hygiene, Epidemiology and Medical Statistics, Medical School, University of Athens, Athens, Greece; The Aaron Diamond AIDS Research Center, 455 First Avenue, New York, NY 10016 USA; School of Biomedical and Healthcare Sciences, Plymouth University, Plymouth, PL4 8AA UK

**Keywords:** Endogenous retrovirus, HERV-K, ERV, XRV, Evolution, Life history traits

## Abstract

**Background:**

Endogenous Retroviruses (ERVs) are retroviruses that over the course of evolution have integrated into germline cells and eventually become part of the host genome. They proliferate within the germline of their host, making up ~5% of the human and mouse genome sequences. Several lines of evidence have suggested a decline in the rate of ERV integration into the human genome in recent evolutionary history but this has not been investigated quantitatively or possible causes explored.

**Results:**

By dating the integration of ERV loci in 40 mammal species, we show that the human genome and that of other hominoids (great apes and gibbons) have experienced an approximately four-fold decline in the ERV integration rate over the last 10 million years. A major cause is the recent extinction of one very large ERV lineage (HERV-H), which is responsible for most of the integrations over the last 30 million years. The decline however affects most other ERV lineages. Only about 10% of the decline might be attributed to an accompanying increase in body mass (a trait we have shown recently to be negatively correlated with ERV integration rate). Humans are unusual compared to related species – Old World monkeys, great apes and gibbons – in (a) having not acquired any new ERV lineages during the last 30 million years and (b) the possession of an old ERV lineage that has continued to replicate up until at least the last few hundred thousand years – the potentially medically significant HERVK(HML2).

**Conclusions:**

The human genome shares with the genome of other great apes and gibbons a recent decline in ERV integration that is not typical of other primates and mammals. The human genome differs from that of related species both in maintaining up until at least recently a replicating old ERV lineage and in not having acquired any new lineages. We speculate that the decline in ERV integration in the human genome has been exacerbated by a relatively low burden of horizontally-transmitted retroviruses and subsequent reduced risk of endogenization.

**Electronic supplementary material:**

The online version of this article (doi:10.1186/s12977-015-0136-x) contains supplementary material, which is available to authorized users.

## Background

Retroviral replication involves integration into a chromosome of the host cell. Over the last 100 million years (my), retroviruses have repeatedly integrated into germline cells of their host and thus become incorporated into the host genome [1]. Such Endogenous Retroviruses (ERVs) can be grouped into families [2], each one representing the subsequent proliferation of an independent infection of the host genome. Each viral integration is referred to here as a locus, and these loci inevitably accumulate mutations at the host background level, gaining frameshifts and premature stop codons that make them replication-deficient. It is only by the continual copying of loci that the family persists through evolutionary time. There are ~100,000 ERV loci in ~50 families (also called groups) in the human genome [3,4] making up ~5% of the total sequence (>8% if the other transposable elements called MaLRs are included) [5].

Despite being pathogenic in other animals, and retroviruses typically being oncogenic, no causal link with human disease has been proven [6,7]. This apparent benignness might be the result of a slow down in the rate of ERV integration. The early genome sequencing projects suggested that in recent evolutionary history ERVs have been much less active, in the sense of producing new loci, in humans than mice [8], and no human ERV locus is known that is capable of replication. For example, no instances of integration within somatic tissues by human ERVs have been observed whereas so-called insertional mutagenesis is a well understood mechanism by which some ERV loci cause cancer in laboratory mice. Whether humans are unusual in their level of ERV integration has not been systematically investigated.

Although published genome sequences are sometimes a composite made by combining different regions from different individuals, each sequence can be thought of as representing the genome of a single (haploid) individual. There is no known mechanism to precisely excise ERV loci, so the genome contains a record of the history of ERV integrations that have drifted to fixation, albeit one where because of recombination each region is randomly drawn from the population. Many loci that are unfixed in the species will not be in the genome sequence, but these loci will have integrated relatively recently, e.g. the mean time for a neutral allele to drift to fixation is ~800,000 years in the ancestral human population [9]. We date the integration of ERV loci using several methods [10]: (i) nucleotide divergence within the locus, (ii) divergence from other loci in the same genome and (iii) the presence or absence of the locus in related host species. Counting the number of integrations that have taken place in each genome during a given time period allows us, if we assume that selection acting on ERVs is similar across species, to then compare ERV integration rates.

Here we measure the rate of ERV integration in the genome of ancestral humans and other catarrhine primates (Old World monkeys, great apes and gibbons). Multiple catarrhine genomes have been sequenced and their ERVs are well characterized, allowing integrations to be dated accurately. We also compare the integration rates across a diverse range of other mammals using a more approximate method that does not require such characterization. We attempt to explain the patterns using viral and host life-history factors that we have shown previously to be correlated with the ERV integration rate [11,12], and examine in detail the family HERVK(HML2) [13], called here HK2 for brevity. This is the only family that has continued to replicate in the human population until at least ~250,000 years ago [14]. The family is important because expression of HK2 proteins is upregulated in a range of diseases, although it is not known whether it is involved in their causality or is a result of their pathophysiology [15-17]. This upregulation also raises the possibility of HK2 loci serving as immunotherapy targets in cancer and HIV therapy [18-20].

We report a steep decline over the last ~10 million years in the ERV integration rate within humans and other hominoids (great apes and gibbons) compared to Old World Monkeys and other mammals. This decline is attributable largely, although not entirely, to the extinction of one very large family (whose replication cycle we have previously shown to be associated with increased proliferation). The HK2 family in humans, however, represents a possibly unique persistence of a replicating family from the origin of the catarrhines. Another feature of the human genome, shared only with the orangutan, is the absence of any new ERV families, and we discuss possible reasons for this.

## Results

### ERV integration rate has declined in humans and other hominoids

**Figure 1:**
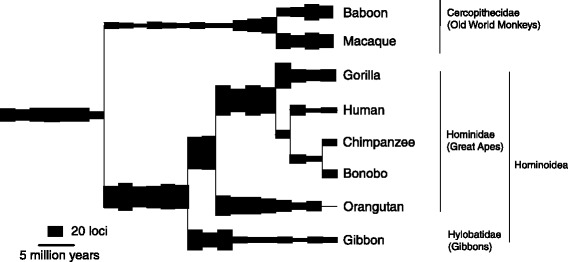
**Rate of ERV integration in the sequenced catarrhines.** Branch thickness shows the number of loci estimated to have integrated at different times, with each increment corresponding to a period of two million years. Integration dates are estimated by LTR divergence (except in the poorly assembled baboon, where they are estimated using a nearest neighbor analysis). Numbers of loci have been normalized using the human genome as a reference to allow for variation in quality of genome assembly as follows: branch thickness leading to human is calculated from the human genome; other branch thicknesses are adjusted proportional to a comparison between (i) the number of loci that integrated into the human genome and (ii) the number that integrated into the second species’ genome during the time period when the genome was shared. The baboon was similarly normalized using the macaque instead of the human genome. Data for each species are shown as frequency histograms in Additional file 1: Figure S1.

We extracted the nucleotide sequences of all ERV loci in the catarrhine genomes and dated the more intact ones by first calculating a rate of nucleotide divergence in representative loci, and then applying this to the divergence between their LTRs (Long Terminal Repeats). The LTRs are regions at either end of the full-length integrated virus (provirus) that are identical at integration but gradually diverge through time with the accumulation of mutations. We see a striking decline in the rate of ERV integration during the last ~10my in the genomes of all sequenced hominoids (great apes and gibbons) but not in the Old World monkeys (Cercopithecidae) (Figure 1). The decline in the human genome since the divergence from the chimpanzee is similar to that of other hominoids, e.g. showing a similar ratio of the number of loci that integrated before and after that event (Table 1). The difference in Table 1 among hominoids can probably be attributed to differing methods and quality of genome sequencing and assembly, e.g. the number of loci in the human, chimpanzee, bonobo and gorilla genomes that are older than 8my should by definition be identical – as until this time they share the same genome – but in our analyses they differ, with the gorilla being particularly low (Additional file 1: Figure S1). Converting the numbers of loci in Table 1 into a rate shows a 73% decline in the human genome over the last 6.6my compared to the preceding 25.0my.

**Table 1:** **Comparison of age of loci among catarrhine genomes**

**Species**	**Number of young loci** ^**a**^ **(0–6.6my)**	**Number of old loci** ^**b**^ **(6.6–31.6my)**	**Ratio of young to old loci**
Human	40	568	0.070
Bonobo	62	589	0.105
Chimpanzee	50	362	0.138
Gorilla	26	197	0.132
Orangutan	13	200	0.065
Gibbon	13	156	0.083
Macaque	76	145	0.524
Baboon^c^	171	633	0.270

^a^Loci estimated to have integrated since the human-chimp divergence, 6.6mya. Age of locus calculated using our paired LTR method with a substitution rate of 1.0x10^−9^ substitutions per nucleotide per year and a Jukes-Cantor correction for multiple hits.

^b^Loci estimated to have integrated between the human-chimp and the human-macaque divergence, 31.6mya.

^c^We could not find the LTRs of most loci in the poorly assembled baboon genome and therefore dated loci using our approximate nearest neighbor method.

**Figure 2:**
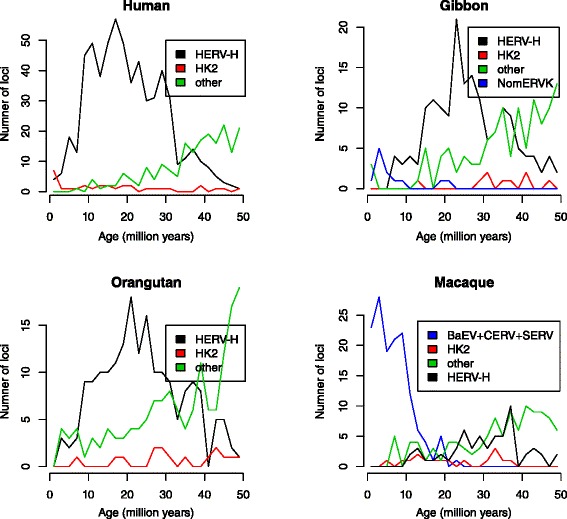
**Age and number of ERV integrations in the human and other representative catarrhine genomes. **Loci analysed were all full-length and dated using LTR divergence.

In Figure 2 we show these changes at the level of individual ERV families and observe the following.

1. The overall changes in the hominoids are dominated by the changes within one family, HERV-H: e.g. it makes up 88% of all the ERV integrations into the human genome within the last 30my. It is the recent extinction of this megafamily, defined as a family that is abnormally large typically as a result of adopting an entirely intracellular replication cycle [11], that produces the steep decline in the overall rate over the last 10my. This decline occurs independently in the separate hominoid lineages: e.g. human, orangutan and gibbon. Most other families also decline, as shown for example by the ‘other’ category in the human plot, which contains another 15 families (the remaining known ERV families are too old and their loci too degraded to have been included here).
2. In the human genome, we see a decline over time in the integration rate of all families except one, HK2 (the apparent recent increase is explained below). This family entered the genome of the ancestral catarrhine 32–44 million years ago (mya), i.e. after the split from the New World monkeys and before the split from the Old World monkeys [21]. Several lines of evidence show this family to have been replicating in the human population up until at least the last few hundred thousand years. However, the sister lineages of HK2 in most other catarrhines appear to have gone extinct. The youngest HK2 locus we found in the chimpanzee was ~3my old, although two loci in the more poorly assembled bonobo genome might be younger. Elsewhere the youngest we found were dated to 4mya (macaque), 7mya (orangutan), 10mya (gorilla) and 13mya (gibbon). Limitations of the baboon genome assembly does not allow loci to be dated using their LTRs but our nearest neighbor method of dating loci reveals no recent HK2 integrations (data not shown).
3. The HK2 family is the only family we found that has continued to replicate since the origin of the catarrhines. All other old families appear to have gone extinct, lacking loci with identical or very similar LTRs and lacking short branches on the dendrograms showing the sequence similarity of ERV loci in individual genomes (Figure 3). We did find occasional loci with identical LTRs from families otherwise represented only by older loci, but we assume these represent either chance identity between short (~100 nucleotide) fragments of LTR or instances of gene conversion (see Methods).

**Figure 3:**
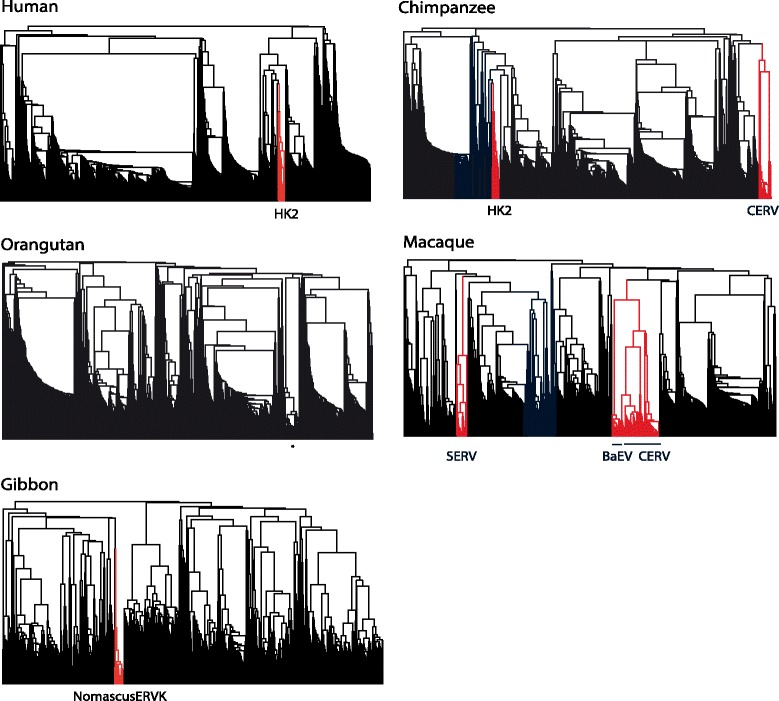
**Dendrogram of loci in selected catarrhines.** Recently copying families plus HK2 in the human and chimpanzee genomes are shown in red. Families such as BaEV show bursts of copying restricted to near the tree tip. For clarity, we excluded loci that had integrated before the origin of the catarrhines. The asterisk in the orangutan shows a clade of loci detected only in unassembled parts of the X chromosome and chromosome 1. These possibly represent loci within repeat regions that have been copied by the host, or assembly errors.
4. Excluding HK2, other recent integrations are all from recently acquired families. In the hominoids, all other integrations within the last two million years come from two families with restricted taxonomic distributions showing that the family is derived from genome infections after the origin of the catarrhines: (i) CERV (Chimpanzee ERV), also known as PtERV (*Pan troglodytes* ERV), was discovered in the genomes of the chimpanzee and gorilla but is not in the human or orangutan genome [22], and (ii) a new type II family we found that was restricted to the gibbon (labelled NomascusERVK in Figure 3). Similarly, although the macaque and baboon genomes have many recently integrated ERV loci, these are all from younger families. In the macaque, we have three families (or groups of closely related families): (i) a close relative of CERV, (ii) BaEV (Baboon Endogenous Virus), which was first described from several baboon species [23], and (iii) SERV (Simian Endogenous type D Retrovirus) [24]. The baboon has the recent families found in the macaque plus its own undescribed type II family (the poor assembly of the baboon genome does not allow us to characterize this family).

### Hominoid decline is greater than in non-hominoid mammals

This decline in the rate of ERV integration in the hominoid genomes is generally greater than that observed in a diverse range of other mammals (Table 2), including other primates. In this reanalysis of data from a previous study [11] (with the addition of the gibbon but not the bonobo), we compare the number of loci estimated to have integrated in similar time periods to those in Table 1. Because the ERVs in many non-catarrhine genomes have not been studied, we had to use both a cruder mining technique, merely recovering a region of the conserved *pol* gene from each locus, and a more approximate method to date the loci using only this information (“nearest neighbor” – see Methods). In the absence of lineage-specific data, we also had to employ a single mammalian nucleotide substitution rate. Considering the catarrhine species, the results of this analysis are poorly correlated to the results of our more detailed analysis shown in Table 1, with up a six-fold difference in the ratio of young to old loci (orangutan). We suspect that there are a number of artifacts here, e.g. the clade of apparently recently integrated loci in the orangutan (Figure 3) that are only in unassembled parts of the X chromosome and chromosome 1. Nevertheless, this second analysis shows the same general trend: the mean ratio of young to old loci in the hominoids was 0.16 (n = 5) compared to a mean of 0.83 in the non-hominoids (n = 34). This difference is significant (Wilcoxon rank sum test, p-value = 0.002) and remains so even if the human genome, with the lowest ratio of young to old loci observed in any mammal, is excluded (P = 0.010).

**Table 2:** **Comparison of age of loci among diverse mammal genomes**

**Species**	**Common name**	**Order**	**Number of young loci (0–6.6my)** ^**a**^	**Number of old loci (6.6-31.6my)** ^**a**^	**Ratio of young to old loci**
*Homo sapiens*	Human	Primates - Hominoidea	66	2468	0.027
*Pan troglodytes*	Chimpanzee	Primates- Hominoidea	152	1745	0.087
*Gorilla gorilla*	Western gorilla	Primates- Hominoidea	138	1345	0.103
*Pongo pygmaeus*	Bornean orangutan	Primates- Hominoidea	723	1857	0.389
*Nomascus leucogenys*	White-cheeked crested gibbon	Primates - Hominoidea	1113	6083	0.183
*Macaca mulatta*	Rhesus macaque	Primates - Cercopithecidae	187	1531	0.122
*Papio hamadryas*	Hamadryas baboon	Primates- Cercopithecidae	241	1266	0.190
*Callithrix jacchus*	Common marmoset	Primates - New World Monkeys	136	605	0.225
*Tarsius syrichta*	Philippine tarsier	Primates - tarsiers	1845	1195	1.544
*Microcebus murinus*	Gray mouse lemur	Primates - lemurs	751	398	1.887
*Otolemur garnettii*	Northern greater galago	Primates - lorises	255	412	0.619
*Echinops telfairi*	Small Madagascar hedgehog tenrec	Afrotheria	185	786	0.235
*Bos taurus*	Domestic cow	Artiodactyla	182	793	0.230
*Sus scrofa*	Domestic pig	Artiodactyla	19	59	0.322
*Vicugna pacos*	Alpaca	Artiodactyla	176	96	1.833
*Ailuropoda melanoleuca*	Giant Panda	Carnivora	56	80	0.700
*Canis familiaris*	Domestic dog	Carnivora	26	119	0.218
*Felis catus*	Domestic cat	Carnivora	167	204	0.819
*Tursiops truncatus*	Bottlenose dolphin	Cetacea	20	192	0.104
*Myotis lucifugus*	Little brown bat	Chiroptera	308	308	1000
*Pteropus vampyrus*	Large flying fox	Chiroptera	106	187	0.567
*Erinaceus europaeus*	West European hedgehog	Erinaceomorpha	1667	1268	1.315
*Procavia capensis*	Cape hyrax	Hyracoidea	1164	870	1.338
*Ochotona princeps*	American pika	Lagomorpha	309	277	1.116
*Oryctolagus cuniculus*	European rabbit	Lagomorpha	316	268	1.179
*Macropus eugenii*	Tammar wallaby	Marsupialia	54	491	0.110
*Monodelphis domestica*	Gray short-tailed opposum	Marsupialia	1162	4344	0.267
*Ornithorhynchus anatinus*	Duck-billed platypus	Monotremata	163	94	1.734
*Equus caballus*	Horse	Perissodactyla	22	159	0.138
*Choloepus hoffmanni*	Hoffmanns two-toed sloth	Pilosa	711	1425	0.499
*Loxodonta africana*	African bush elephant	Proboscidea	416	1411	0.295
*Cavia porcellus*	Guinea pig	Rodentia	1608	1450	1.109
*Dipodomys ordii*	Ord's kangaroo rat	Rodentia	273	223	1.224
*Mus musculus*	House Mouse	Rodentia	3045	1402	2.172
*Rattus norvegicus*	Brown rat	Rodentia	737	897	0.822
*Spermophilus tridecemlineatus*	Thirteen-lined ground squirrel	Rodentia	1271	726	1.751
*Tupaia belangeri*	Northern treeshrew	Scandentia	295	315	0.937
*Sorex araneus*	Common shrew	Soricomorpha	502	395	1.271
*Dasypus novemcinctus*	Nine-banded armadillo	Xenarthra	1631	3240	0.503

^a^Age of locus is estimated using our nearest-neighbor method with a substitution rate of 2.2×10^9^ substitutions per nucleotide per year and a Jukes-Cantor correction for multiple hits.

### Confirming that HK2 is an exception to the general hominoid decline

Figures 1 and 2 include only those ERV loci that have retained both of their LTRs, but most ERV loci are represented by a relic structure called a solo LTR. A solo LTR is formed by a crossover between the two LTRs that leads to the excision of the entire internal (protein-coding) region, leaving only a chimeric structure containing the LTR regions that lay outside of the crossover. There is no known mechanism to precisely excise a solo LTR, so we were able to confirm the results of the above analysis as follows. We first extracted the genomic regions that flanked loci in the human genome, and then searched for these flanking regions in other catarrhine genomes. This allowed us to determine whether or not the homologous ERV locus was present or absent. Using this procedure we found 54 HK2 loci (including both full-length and solo LTRs) in the human genome that were absent in the macaque (i.e. represented by the pre-integration site) and which we could score as either present or absent in the chimpanzee (Table 3). For each of these loci we therefore know whether they integrated either (a) after the divergence of human from macaque but before the divergence from chimpanzee (6.6-31.6mya), or (b) after the divergence of human from chimpanzee (<6.6mya). The proportion of the 54 loci that fall into these two groups is very close to that predicted if we assumed a constant rate of integration during our entire 31.6my time period. In contrast, we find a decline among the other ERV families – treated here as one group – since the divergence from the chimpanzee. Although the sample size is small, the difference is significant (Fisher’s Exact Test; P < 0.01). This analysis also suggests that the apparent recent increase in HK2 integration rate in Figure 2 is an artifact caused by only considering full-length loci. We expect a higher proportion of recently integrated loci to be full-length because they have had less time in the genome than old loci in which to undergo recombination and form solo LTRs (and thus be lost to our main analysis). The rate of this recombinational deletion in HK2 decreases with age [25] but that will only affect the magnitude and not the direction of the bias. It has also been observed in multiple mice ERV lineages that the proportion of loci represented by full-length proviruses (rather than by solo LTRs) is higher among more recent integrations [26]. That study showed that some ERV integrations have been deleterious, and this might have selected for the process of recombinational deletion.

**Table 3:** **Comparison of age of HK2 and other loci in the human genome**

**Time of integration**	**ERV family**	
	**HK2**	**Other**
Macaque to chimpanzee^a^	42 (43)^c^	65 (54)^c^
After chimpanzee^b^	12 (11)^c^	3 (14)^c^

^a^Number of loci that integrated after the divergence from the macaque (31.6mya) but before the divergence from the chimpanzee (6.6mya). All loci represented by the pre-integration site in the macaque (or baboon).

^b^Number of loci that integrated after the divergence from the chimpanzee (6.6mya). Loci represented by the pre-integration site in the chimpanzee.

^c^The number shown in parenthesis is that expected if a single integration rate was applied to both time periods.

## Discussion

Our analysis shows a steep decline over the last 10my in the rate of ERV integration in the genomes of human and other hominoids, which might account at least in part for the absence of proven pathogenicity of ERVs in humans. What has caused this decline? We have previously shown that two traits, one host and one viral, are correlated with the ERV integration rate: smaller mammals tend to have more ERVs [12] and loss of the *env* gene leads to greater replication of ERV families [11]. Below we consider in turn the evidence for the possible involvement of these two traits in the observed decline.

Analysing data from the Katzourakis et al. study [12] shows that a doubling in body mass is associated with an approximate 10% reduction in ERV number. Fossil evidence shows that the human lineage has increased in body mass over our time period. The earliest fossil catarrhine species, proconsuloids from the early Miocene (23-16mya), varied greatly in body mass, ranging from an estimated 5 to 75 kg [27], but none of the fossil species from later periods that may represent ancestors of the great apes are as small as the smallest proconsuloids – the 17 kg for a specimen of *Nacholapithecus kerioi* from 15mya [28] being the smallest body mass estimate we can find. The more recent fossil species that may be directly ancestral to humans are all at least 33 kg [29]. From the origin of the great apes we observe therefore at most a five-fold increase in mass, which – even assuming a causal link behind the association – would only lead to a decrease in ERV number of approximately 25% rather than the observed four-fold decrease (Figure 1). We also observe a marked decline in ERV integration in the small-bodied gibbon.

The decline of the ERV integration rate in the hominoid lineage can be chiefly attributed to the gradual extinction during this time period of the HERV-H family. The domination of the ERV community in mammals by a few such megafamilies is typically achieved by the degradation and loss of the viral attachment gene (*env*), with an inferred switch to an entirely intra-cellular life cycle and increase in integration rate [11]. It has been suggested that in HERV-H this pattern is complicated by *in trans* complementation [6]. More reconstruction of the past replication method in this family might reveal additional viral factors than could explain the scale of the decline in ERV integration in hominoids. Similarly, when more mammal genomes are available, larger comparative analyses might identify changes in hominoid biology (in addition to body size) that are associated with reduced ERV integration rate.

In contrast, the small *env*-containing family, HK2, may have continued to replicate exclusively in humans. The apparent extinction of the sister lineage of HK2 in the macaque is significant as this lineage is being investigated as a model for testing a possible ERV-based immunotherapy for HIV in humans [20,30,31]. The extinction explains why other studies have reported finding only a few full-length ORFs in this family [20,30]. An earlier bioinformatic study [32] reported finding HK2 loci in the macaque that had identical LTRs, suggesting they were very recent integrations, but we cannot confirm the existence of such loci.

ERV families do not appear to be able to maintain themselves indefinitely and eventually die, ceasing to create new loci and with their existing loci eventually losing the ability to replicate as they accrue mutations. The birth of new families is therefore essential for the persistence of ERV replication in host genomes. Part of the overall decline of ERV integration in the human genome is the absence of any new families being acquired during the 32my history of the catarrhines. All the analyzed non-human catarrhines except the orangutan have acquired at least one new ERV family. As previously mentioned, ERV families are assumed to be derived from an initial infection of the germline by an exogenous (horizontally-transmitted) retrovirus (XRV), and ERVs can be viewed as a fossil record of retroviral activity through time [6]. The striking decline of the ERV integration rate in humans could thus be due to a lower risk of XRV infection (horizontal transmission) as well as a lower level of genomic replication (vertical transmission). Is there evidence for a lower XRV load in humans compared to, for example, other catarrhines? While the XRV load in humans is well known, data on wild catarrhines are patchy and we are not aware of any systematic comparison of XRV burden. Nevertheless our examination of the literature suggests that humans do have an unusually low XRV load. There are only two human XRVs (HIV and HTLV), both globally at less than 1% prevalence; in contrast, XRVs such as the foamy (SFVs), immunodeficiency (SIVs) and T-lymphotropic viruses (STLVs) appear to be common in non-human catarrhines and typically at higher prevalence [33]. The viral lineage from which the various HIVs are derived, called SIV, although not ubiquitous in catarrhines is often found at a much higher prevalence. For example, the long study of the Central Chimpanzee subspecies (*P. t. troglodytes*), from which HIV-1 was acquired, in the Gombe National Park, Tanzania, found the prevalence to fluctuate between 9 and 18% [34]; other studies found prevalence of 13% in Eastern Chimpanzees (*P. t. schweinfurthii)* [35] and 2% in gorillas [36], both figures estimated from analyses of over 2500 fecal samples. STLV, the relative of HTLV, is also common, with studies showing prevalences of, for example, 14% in gorillas [37], 48% in chimpanzees [38], and in 44% of individuals from seven other primate species sampled as bushmeat in Gabon [39]. A clearer picture emerges from the third common catarrhine virus, SFV (simian foamy virus). This has been found in many catarrhines and, although spillovers of SFVs into humans have been well documented, there is no evidence of persistent human-to-human transmission. Although most data on SFV come from captive populations (where it is very common), some studies show high prevalence in the wild, e.g. being found in 3 of 6 captive but wild-born gibbon (*Nomascus leucogenys*) and 20 of 20 captive but wild-born orangutan [40], in 5 of 27 wild-caught gorillas [41], and 44-100% of 724 chimpanzee fecal samples [42].

The above data are just a snapshot of XRV infection today. However, there is evidence of long evolutionary associations between XRVs and some non-human catarrhines. While HIV is new and pathogenic in humans, SIVs often show remarkably low pathogenicity for their host [43] and have congruent phylogenies with their hosts [44], both observations suggesting long co-existence. That SIV is pathogenic in chimpanzees is thought to reflect their recent acquisition from prey monkeys, and the utility of SIV-infected macaques as a model for HIV infection in humans reflects the absence of SIVs in wild macaques. Similarly, SFVs are also commonly non-pathogenic with congruent host and virus phylogenies [45]. We note that the pattern in T-lymphotropic viruses is more complex: they do not appear to form congruent phylogenies with their hosts [46] and there is no consensus about the date of origin of the various forms of HTLV [47].

If there is indeed an unusually low XRV burden in humans, this may reflect a reduced risk of retroviral transmission. As humans evolved, their behavior changed into having fewer encounters with blood (a major transmission route for XRVs) compared to other primates, either via predation or male-male conflict [48] – a trend that has possibly been reversed in the last century or so with alarming consequences for viral infection [49]. An alternative could be that a persistent endogenous retrovirus such as HK2 might have protected humans from exogenous retrovirus infection, e.g. in Jaagsiekte sheep retrovirus some endogenous loci protect the host from the related but more pathogenic exogenous form, both by receptor competition and blocking capsid trafficking [50]. Receptor competition has been observed *in vitro* for other closely related pairs of ERVs and XRVs [51], but whether it could defend a host from more distantly related XRVs is unknown. There is, however, some *in vitro* evidence that HK2 might interfere in HIV replication by competition for Gag-binding factors [52]. Whatever the cause, having fewer XRVs would reduce the likelihood of endogenization and thus explain the unusual absence of new ERV families in the human genome.

Another way in which ERV and XRVs could interact is by applying selection to innate immunity genes. The APOBEC3 gene family has been shown to have hypermutated at least two HK2 loci in the human genome and is also restrictive against two functionally reconstituted ERVs: HK2 and CERV-1 [53-55]. The role of another restriction factor, TRIM5α, on the replication of ERVs is controversial with one study finding restriction of a functionally reconstituted CERV-1 [56] while another study did not [55]. In the former, Kaiser et al. further reported mutually exclusive restriction of CERV-1 (= PtERV1) and HIV-1 by the TRIM5α of a range of other catarrhines. They speculated that past selection on TRIM5α to protect humans from infection by CERV-1 (= PtERV1) might in part be responsible for our current susceptibility to HIV-1. We are only beginning to understand how ERVs, XRVs and our innate immune system have interacted with each other through evolutionary time [57]. An evolutionary trade-off in the detection of cDNA involving these players (plus other endogenous retroelements) and the risk of autoimmune disease has also just been proposed [58]. Further comparative studies into the role that environmental and immunological factors have played in determining ERV load might help reveal how our immune system controls both vertical and horizontal transmission and determines the lifetime risk of retrovirus-associated disease.

## Conclusions

1. The hominoid genome has undergone a possibly unique collapse of ERV integration in recent evolutionary history compared to that of Old World monkeys and other mammals.
2. Most of this decline is attributable to the recent extinction of one abnormally large family, HERV-H.
3. Another contributing factor to the decline within the human genome is the absence of any new endogenous retroviral lineages acquired in recent evolutionary history. This is unusual among catarrhines.
4. Only a small part of this overall decline can be explained by changes in the one life-history trait – body size – known to be correlated with ERV integration rate.
5. Humans appear to be unique among our catarrhine relatives in the possession of an old ERV family that has continued to replicate up until at least the last few hundred thousand years – the potentially medically significant HK2.

## Methods

### Mining and dating

We probed the following catarrhine genome sequences with the genomic regions (LTR, leader, *gag, pro, pol, env*) of each ERV family: human (*Homo sapiens*), chimpanzee (*Pan troglodytes*), bonobo (*Pan paniscus*), gorilla (*Gorilla gorilla*), orangutan (*Pongo pygmaeus*), Northern white-cheeked gibbon (*Nomascus leucogenys*), rhesus macaque (*Macaca mulatta*) and hamadryas baboon (*Papio hamadryas*). Our library of probes was based on the well-studied human ERV families but supplemented with examples from all non-human families found in the other catarrhines. These non-human families were either already described in the literature – CERV, SERV and BaEV [22-24] – or discovered by us among novel sequences recovered from our earlier mining using the conserved Reverse Transcriptase domain of *pol* [11]. We were thus able to extract full-length loci, defined here as loci with at least 100 nucleotides of both 5′ and 3′ LTR and at least some internal region. This allowed us to date the integration using nucleotide divergence between the LTRs of the proviruses. Ranging in size from 300 to 1000 nucleotides, the two LTRs form the flanks of a provirus (the complete integrated DNA form of a retrovirus) and are identical at the time of integration, accumulating substitutions at the host background rate. Rather than use published estimates of background rate we estimated the substitution rate within the ERVs to be 1.0x10^−9^ per site per year directly using orthologous loci (see below). We then used this rate to convert the nucleotide divergence between the paired LTRs of each locus into a date of integration, correcting for multiple hits using the Jukes-Cantor model.

One drawback with this approach is gene conversion (recombination between the two LTRs of a provirus), which can homogenize the two LTRs leading to an old locus appearing to be younger. The effect of this is relatively minor: e.g. it has been estimated that ~6% of orthologous loci in the macaque and human genomes have undergone some gene conversion [59]. Recombination can also take place between the LTRs of different loci, which has the effect of artificially aging them, but this also affects only a small minority of loci [3].

The ERVs of most non-catarrhine mammal species are poorly studied, so it was not possible to identify the LTRs of all full-length loci. We were therefore compelled to estimate the age of a locus based on its divergence from the most similar other ERV locus in the same genome (a “nearest neighbor” analysis). For this we used sequences from our previous *in silico* screen of mammalian genomes [11], which recovered 600 nt long *pol* sequences. For each such *pol* sequence, we calculated the nucleotide divergence from the most similar other locus in the same genome, and then converted this to an integration date correcting for multiple hits using the Jukes-Cantor model. To estimate the integration rate we applied to all genomes a uniform mammalian nucleotide substitution rate, derived from neutral nuclear protein-coding sites, of 2.2×10^−9^ per site per year [60]. This study found rates to be broadly similar across different mammalian lineages, e.g. the average rate difference between primates and rodents was less than 10%. While the quality of assembly of these non-catarrhine genomes varies greatly, making comparison of absolute numbers across genomes problematic, our conversion to a ratio of the number of young loci to the number of old loci should reduce the error caused by this source of variation.

### Building dendrograms

For each genome, a matrix was made of all pairwise dissimilarities between the nucleotide sequences using the EMBOSS *water* program [61], an implementation of the Smith-Waterman alignment algorithm (with gap opening and extension penalties of 10 and 4 respectively). After excluding loci that did not have a 300 nucleotide long match of at least 90% sequence identity with at least one other locus (removing loci that would have integrated before the platyrrhine/catarrhine split) we then built a dendrogram using UPGMA in R [62].

### Estimating rate of substitution

By finding synteny of the pre-integration sites (i.e. homology of the host genome sequences adjacent to the LTRs), we identified six full-length orthologous loci from three ERV families (HERV-H, HERV-L, and HK2) in at least four of the following species: human, chimpanzee, gorilla, macaque and gibbon. For each set of orthologous loci we built an alignment and ran a molecular clock analysis with BEAST [63,64] for at least 10^6^ generations (Estimated Sample Size > 200). We used the General Time Reversible substitution model with a gamma distribution to account for variation of the rate among sites (GTR + G) [65] and an uncorrelated log-normal relaxed molecular clock model [66]. We used the previously reported times to Most Recent Common Ancestor (tMRCA) of the other catarrhines to human to calibrate the molecular clock [67]: chimpanzee: 6.60 (5.40-7.96) mya; gorilla: 8.30 (6.58-10.07) mya; orangutan: 16.52 (13.45-19.68) mya; gibbon: 20.32 (16.59- 24.22) mya; macaque: 31.56 (25.66-37.88) mya.

The median rate on branches of these trees was found to be between 0.6 and 1.3x10^−9^ substitutions per site per year, with an overall mean of these rates being 1.0×10^−9^. Other studies using several homologous LTRs in different families have found similar rates: between 1.0×10^−9^ and 1.3×10^−9^ [68]. A rate of ~1×10^−9^ was also found for non-coding genome regions among catarrhines [69]. The Subramanian and Kumar study [69] concluded that the two-fold higher substitution rate among four-fold degenerate sites across all mammals [60] was probably due to differences in the abundance of CG dinucleotides in coding and non-coding regions.

### Estimation of effect on ERV integration of body mass change

Our analysis of the relationship between (logged) number of ERV loci and (logged) host body mass shows a slope of −0.155 [12]. This suggests that, for example, a doubling in body mass leads to decrease in ERV number of 10%. Humans have a current mean body mass of 59 Kg, with great apes ranging from bonobo (35 Kg) to gorilla (114 Kg) [70], and, even if the body mass at the origin of the great apes was only one-fifth of this (less than the smallest fossil estimate we can find [28]), the increase in body mass would lead to reduction in ERV number of less than 25%.

## Additional file

Additional file 1: Figure S1. Frequency histograms of loci used to construct Figure 1. Ages of loci are estimated using divergence between the two LTRs except for the poorly assembled baboon genome, where ages were estimated using nearest neighbor analysis (hence also the larger number of loci recovered).

## Abbreviations

my: Million years
mya: Million years ago
ERV: Endogenous retrovirus
HERV: Human endogenous retrovirus
LTR: Long terminal repeat
CERV: Chimpanzee ERV
PtERV: Pan troglodytes ERV
BaEV: Baboon endogenous virus
SERV: Simian Endogenous Type D Retrovirus
ORF: Open reading frame
XRV: Exogenous retrovirus
SFV: Simian foamy virus
SIV: Simian immunodeficiency virus
HIV: Human immunodeficiency virus
STLV: Simian T-lymphotropic virus
HTLV: Human T-lymphotropic virus
UPGMA: Unweighted pair group method with arithmetic mean
EMBOSS: European molecular biology open software suite
tMRCA: Time to most recent common ancestor
BEAST: Bayesian evolutionary analysis sampling trees
