# Supplementary material for: The decline of human endogenous retroviruses: extinction and survival
Source: Retrovirology. 2015 Feb 2;12:8. doi: 10.1186/s12977-015-0136-x (PMC4335370; doi:10.1186/s12977-015-0136-x)
Supplement: Additional file 1: Figure S1. — Frequency histograms of loci used to construct Figure 1. Ages of loci are estimated using divergence between the two LTRs except for the poorly assembled baboon genome, where ages were estimated using nearest neighbor analysis (hence also the larger number of loci recovered). [file 12977_2015_136_MOESM1_ESM.pdf]

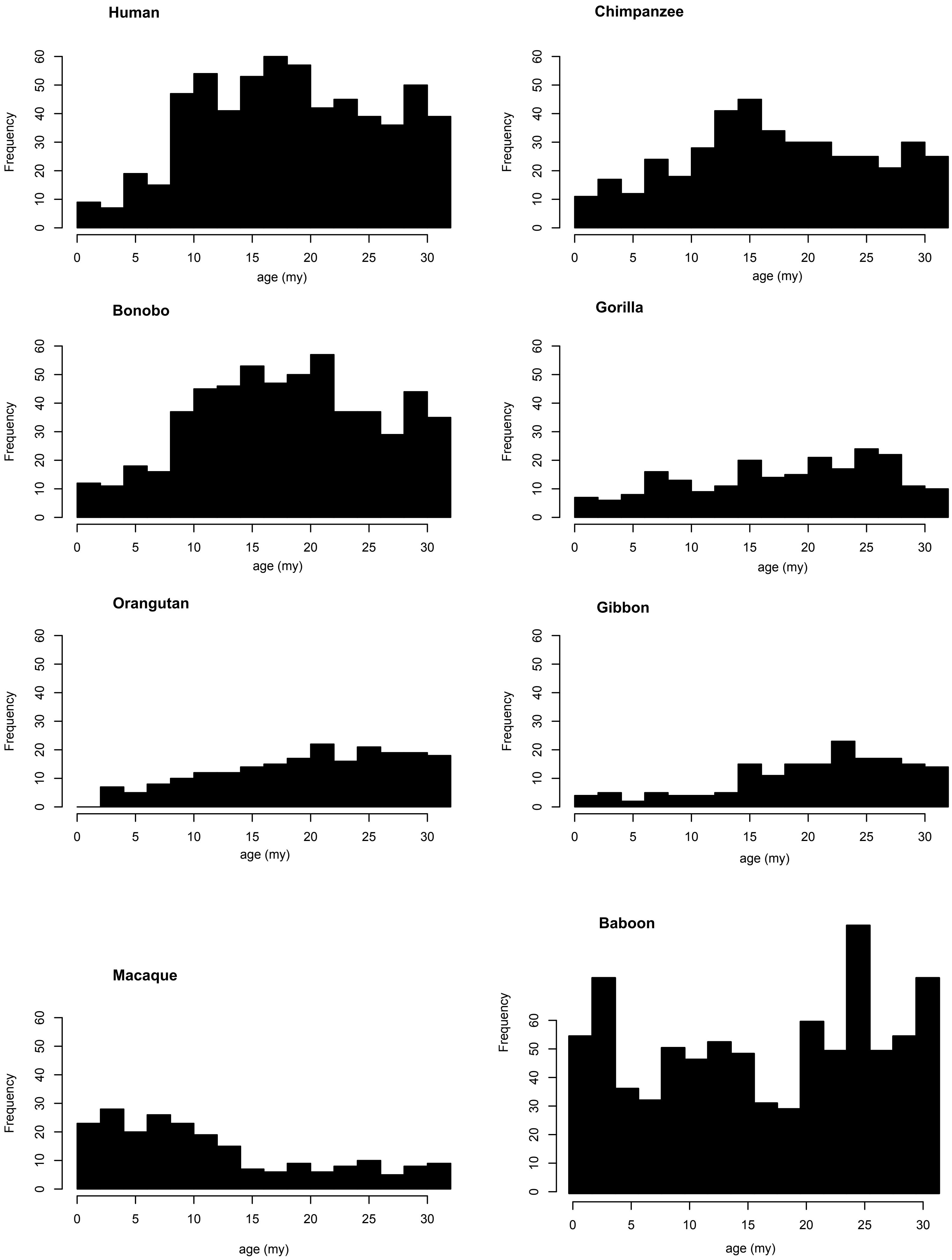

Figure S1. Frequency histograms of loci used to construct Figure 1. Ages of loci are estimated using divergence between the two LTRs except for the poorly assembled baboon genome, where ages were estimated using nearest neighbor analysis (hence also the larger number of loci recovered).
